# Exogenous hemin improves Cd^2+^ tolerance and remediation potential in *Vigna radiata* by intensifying the HO-1 mediated antioxidant defence system

**DOI:** 10.1038/s41598-021-82391-1

**Published:** 2021-02-02

**Authors:** Lovely Mahawar, Robert Popek, Gyan Singh Shekhawat, Mohammed Nasser Alyemeni, Parvaiz Ahmad

**Affiliations:** 1 Plant Biotechnology and Molecular Biology Laboratory, Department of Botany, Centre for Advanced Studies, Jai Narain Vyas University, Jodhpur, Rajasthan 342001 India; 2 Section of Basic Research in Horticulture, Department of Plant Protection, Institute of Horticultural Sciences, Warsaw University of Life Sciences-SGGW (WULS-SGGW), Nowoursynowska 159, 02-776 Warsaw, Poland; 3 Botany and Microbiology Department, College of Science, King Saud University, Riyadh, 11451 Saudi Arabia

**Keywords:** Physiology, Plant sciences

## Abstract

The present study evaluated the effects of exogenous hemin on cadmium toxicity in terms of metal accretion and stress resilience in *Vigna radiata* L. (Wilczek). One-week-old seedlings were treated with CdCl_2_ (50 μM) alone and in combination with hemin (0.5 mM) in half-strength Hoagland medium for 96 h. The optimum concentrations of Cd and hemin were determined on the basis of haem oxygenase-1 activity. The results demonstrated that under Cd stress, plants accumulated a considerable amount of metal in their tissues, and the accumulation was higher in roots than in leaves, which significantly reduced the plant biomass and chlorophyll content by increasing the oxidative stress (MDA and H_2_O_2_ content). However, hemin supplementation under Cd,-stress improved plant growth by enhancing the harvestable biomass and photosynthetic pigments, increasing antioxidant activities (SOD, APX, POD, HO-1 and proline), lowering oxidative damage and increasing Cd tolerance in plants. Furthermore, the application of hemin enhances the removal efficiency of Cd in *V. radiata* by increasing the uptake of Cd via roots and its translocation from roots to foliar tissues. Thus, the study suggests that hemin has the potential to improve the stress tolerance and phytoremediation ability of heavy metal-tolerant plants so that they can be used instead of hyperaccumulators for remediation of Cd-contaminated environments.

## Introduction

Heavy metal contamination is one of the key environmental issues, particularly for agricultural areas, due to the continuous increase in human activities, such as intense usage of phosphate fertilizers, swift urbanization, industrialization and increased utilization of sewage sludge, that accelerate the discharge of noxious elements into the surroundings^1,2^. Among heavy metals, cadmium (Cd) is the most dangerous environmental pollutant that severely restricts the growth and development of plants^3^. Cadmium is readily taken up by plant roots and transferred to foliar tissues, where it starts to accumulate in the esculent parts. This facilitates the entry of Cd into the food chain when consumed by living organisms and causes severe threats to their health^3–5^. Moreover accrued Cd in crop tissues results in diverse structural, biochemical and physiological transformations. Cd^2+^ disturb the nutrients and water uptake in plants, hinder opening and closing of stomata, inhibits the activity of Calvin cycle enzymes (particularly RUBISCO), affects photosynthesis [via hampering photoactivation of photosystem II (PS II)], respiration, carbohydrate metabolism, alters antioxidants activity and declines the yield and productivity of crop plants^6^. Therefore, an efficient approach for Cd remediation from contaminated environments is essential to prevent severe damage in plants as well as to inhibit Cd accumulation in edible plant tissues. Conventional physical and chemical remediation techniques are costly and lead to secondary contamination of the surrounding areas. Conversely, phytoremediation is a promising green approach that utilizes the ability of hyperaccumulator crops,- to take up pollutants from contaminated environments^7^. Phytoremediation has been gaining importance as an efficient, economically feasible, eco-friendly and highly stable technology^7^. However, hyperaccumulators are not particularly abundant, and their slow growth rate as well as reduced biomass limits their phytoremediation efficiency^3^. Therefore, an alternative approach is needed that strengthens the phytoremediation efficiency of metal-tolerant plants so that they can be used as an efficient biological tool to remediate heavy metal pollution. Several chemical reagents and bioregulators, such as ethylene, melatonin, nitric oxide and plant growth regulators (PGRs), are known to enhance Cd stress tolerance by modulating various physiochemical processes that improve crop biomass and further stimulate the uptake and translocation of heavy metals^2,8,9^.

Numerous reports have demonstrated that the application of hemin as a potent biostimulator mitigates metal-induced oxidative stress by enhancing the stress tolerance of plants^1,10^. For example, the application of exogenous hemin ameliorates Al and Cd toxicity in *Medicago sativa* L.^11,12^, induces ammonium tolerance in *Oryza sativa* L.^10^ and enhances salinity stress adaptations in *Triticum aestivum* L.^13^. Hemin, chemically known as ferroprotoporphyrin IX, is a derivative of haem that shares similar chemical properties with natural haem. Since hemin acts as haem, it greatly enhances the catalysis of the haem oxygenase-1 enzyme by overexpressing its mRNA and increasing protein abundance; hence, hemin is regarded as a proficient activator of haem oxygenase-1 that exerts protective effects against various abiotic stresses in an enzyme-dependent manner^5^. Therefore, it is possible to implicate hemin as an effective and efficient biostimulator to improve the phytoremediation potential of heavy metal-tolerant plants.

Mung bean (*Vigna radiata* L. Wilczek) is an eco-friendly traditional legume that is consumed by the majority of the human population, particularly in Asia and Africa, after cereals^14^. It is a good source of proteins, vitamins and minerals and serves to provide a balance in diets mostly composed of lysine-deficient cereal grains^15^. Moreover, *V. radiata* has high tolerance against metals and can gather a substantial quantity of chromium, cadmium, copper, zinc and nickel in its tissues when subjected to polluted environments^16,17^. Hence, it could be advantageous to study the enhancement of this crop’s potential for cadmium remediation. To the best of our knowledge, the influence of hemin on the cadmium resilience and removal potential of *V. radiata* has not yet been evaluated. Therefore, the aims of the present work were (1) to investigate the effect of exogenous hemin on the biomass production and chlorophyll content of *V. radiata*; (2) to analyse the mechanism by which hemin affects Cd tolerance of *V. radiata*; and (3) to verify the influence of exogenous hemin in improving the Cd phytoremediation efficiency of *V. radiata*.

## Results

### Impact of Cd and hemin on crop morphology

**Figure 1:**
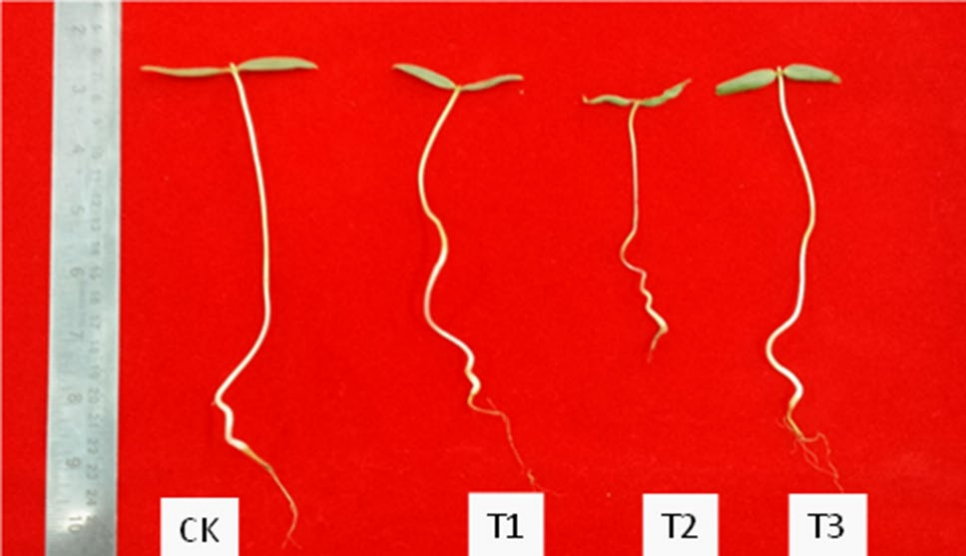
Effect of hemin (0.5 mM) on morphology of *Vigna radiata* seedlings treated with CdCl_2_ (50 μM) for a period of 96 h.

The length and biomass of *V. radiata* seedlings were studied to evaluate the adverse impact of cadmium on crops and the role of hemin against Cd toxicity. In the current work, cadmium negatively affected plant morphology. Compared with the control, the plant biomass (fresh and dry weight) in the Cd-treated plants decreased by 33.33% and 42.86%, respectively, whereas the dose of hemin, under Cd stress conditions (T3), recovered the biomass by 1.17- (fresh weight) and 1.13 (dry weight)-fold more than that under the Cd treatment (T2) (Table 1). Similar trends were observed for plant height and the tolerance index. A 22.86% increase in root length and 24.14% increase in shoot length was noted when seedlings of *V. radiata* were exposed to the combined treatment of hemin + Cd compared with the Cd treatment alone (Fig. 1). The tolerance index decreased by 33.3% under Cd stress, which improved by 17.2% under an exogenous supply of hemin. However, the improvements in biomass and height of the plants in the combined treatment were lower than those of untreated seedlings (Table 1).

**Table 1:** Effect of an exogenous hemin (0.5 mM) on biomass, height, tolerance index, leaf water content and chlorophyll (Chl a, Chl b and Total Chl) content in seedlings of *V. radiata* treated with CdCl_2_ (50 µM) for a period of 96 h.

Treatments	Fresh weight (g)	Dry weight (g)	Root length (cm)	Shoot length (cm)	Tolerance index (%)	Leaf water content (%)	Chlorophyll a (mg g^−1^ Fwt of tissue)	Chlorophyll b (mg g^−1^ Fwt of tissue)	Total chlorophyll (mg g^−1^ Fwt of tissue)
CK	1.56 ± 0.02a	0.14 ± 0.001a	10.4 ± 0.25a	7.8 ± 0.61ab	100 ± 0.00a	91.0 ± 8.25b	0.34 ± 0.002c	0.37 ± 0.004c	0.71 ± 0.03c
T1	1.55 ± 0.01a	0.13 ± 0.001a	10.1 ± 0.38a	8.4 ± 0.02a	99.4 ± 4.52b	91.6 ± 12.2b	0.36 ± 0.002b	0.47 ± 0.002a	0.83 ± 0.01a
T2	1.04 ± 0.01c	0.08 ± 0.002b	7.0 ± 0.12b	5.8 ± 0.04c	66.7 ± 5.21d	92.3 ± 6.32a	0.28 ± 0.004d	0.25 ± 0.001d	0.53 ± 0.01d
T3	1.22 ± 0.01b	0.09 ± 0.005b	8.6 ± 0.06ab	7.2 ± 0.15b	78.2 ± 6.62c	92.6 ± 9.25a	0.39 ± 0.001a	0.42 ± 0.001b	0.80 ± 0.04b

Values are mean ± SE (n = 3) from three replicates for each treatments. Values marked with different letters shows significant differences (p ≤ 0.05) within treatment. Here CK, T1, T2 and T3 indicate control, 0.5 mM hemin, 50 μM CdCl_2_ and 50 μM CdCl_2_ + 0.5 mM hemin respectively.

### Chlorophyll contents

The level of photosynthetic pigments (chlorophyll a, chlorophyll b and total chlorophyll) decreased markedly under cadmium stress. In comparison to the control, decreases of 18.8% (chlorophyll a), 32.3% (chlorophyll b) and 25.7% (total chlorophyll) were recorded when crop seedlings were subjected to cadmium stress. The exogenous application of hemin increased the chlorophyll content under both control and stress conditions. Under control conditions, the hemin treatment (T1) increased the photosynthetic pigments by 1.03 (Chl a), 1.29 (Chl b) and 1.16 (total Chl) times that of the control. Moreover, the cotreatment of 50 µM Cd and 0.5 mM hemin elevated the chlorophyll contents by 1.4 (Chl a), 1.68 (Chl b) and 1.52 fold in comparison with the respective Cd-only treatments (Table 1).

### ROS generation and oxidative damage

**Figure 2:**
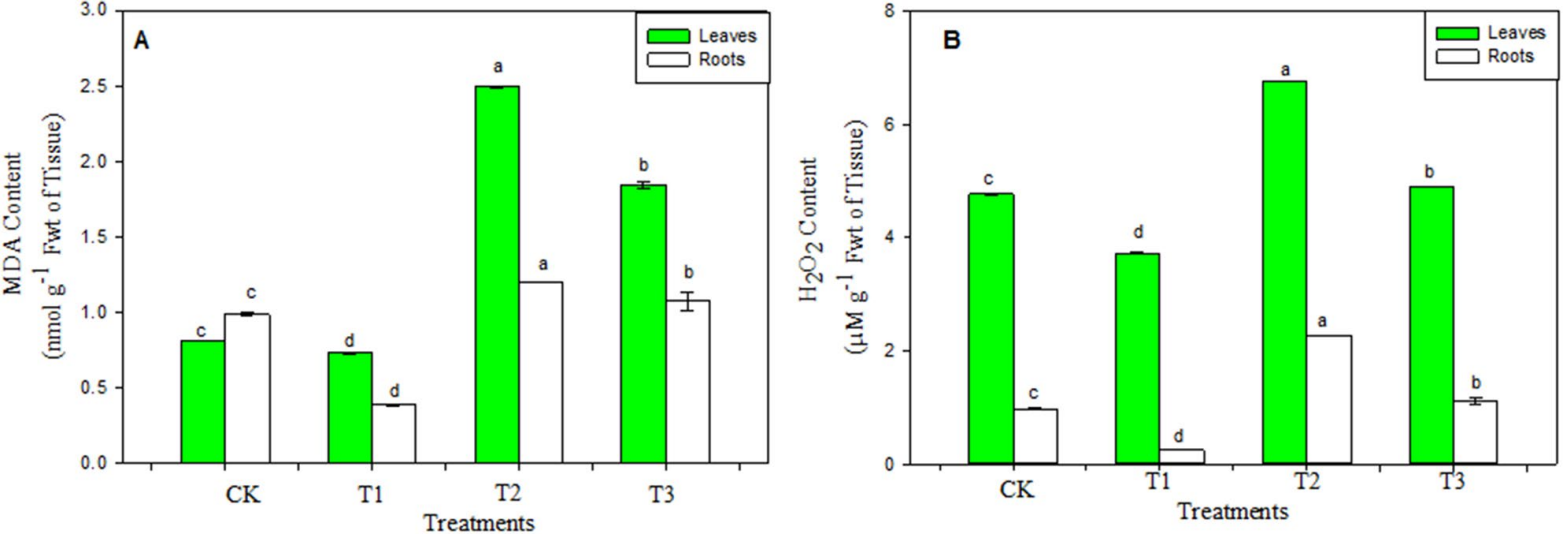
Effect of CdCl_2_ (50 μM) alone or in combination with hemin (0.5 mM) on MDA (A) and H_2_O_2_ (B) content in seedlings of *Vigna radiata*. CK, T1, T2 and T3 indicate control, 0.5 mM hemin, 50 μM CdCl_2_ and 50 μM CdCl_2_ + 0.5 mM hemin respectively. Values are mean ± SE (n = 3) and are statistically significant according to DMRT test (p < 0.05). Data points marked with the different letters show significant differences (p < 0.05) within treatments.

Cadmium addition to the liquid medium, increased H_2_O_2_ production and oxidative damage in plants. The malondialdehyde content (indicator of membrane damage) was found to increase under Cd stress, with levels 3.05 (leaves) and 1.22 (roots) times than those of untreated seedlings (Fig. 2A). Moreover, there was a 41.6% (leaves) and 130.9% (roots) rise in the H_2_O_2_ content of *Vigna radiata* seedlings treated with 50 µM CdCl_2_ (Fig. 2B). The increase in H_2_O_2_ was responsible for the rise in lipid peroxidation (LPX). However, the application of hemin reduced both the MDA (25.9% in leaves and 10. 7% in roots) (Fig. 2A) and H_2_O_2_ (by 27.5% in leaves and 50.4% in roots) (Fig. 2B) contents under CdCl_2_ compared to those cadmium-treated plants without hemin.

### Proline content

**Figure 3:**
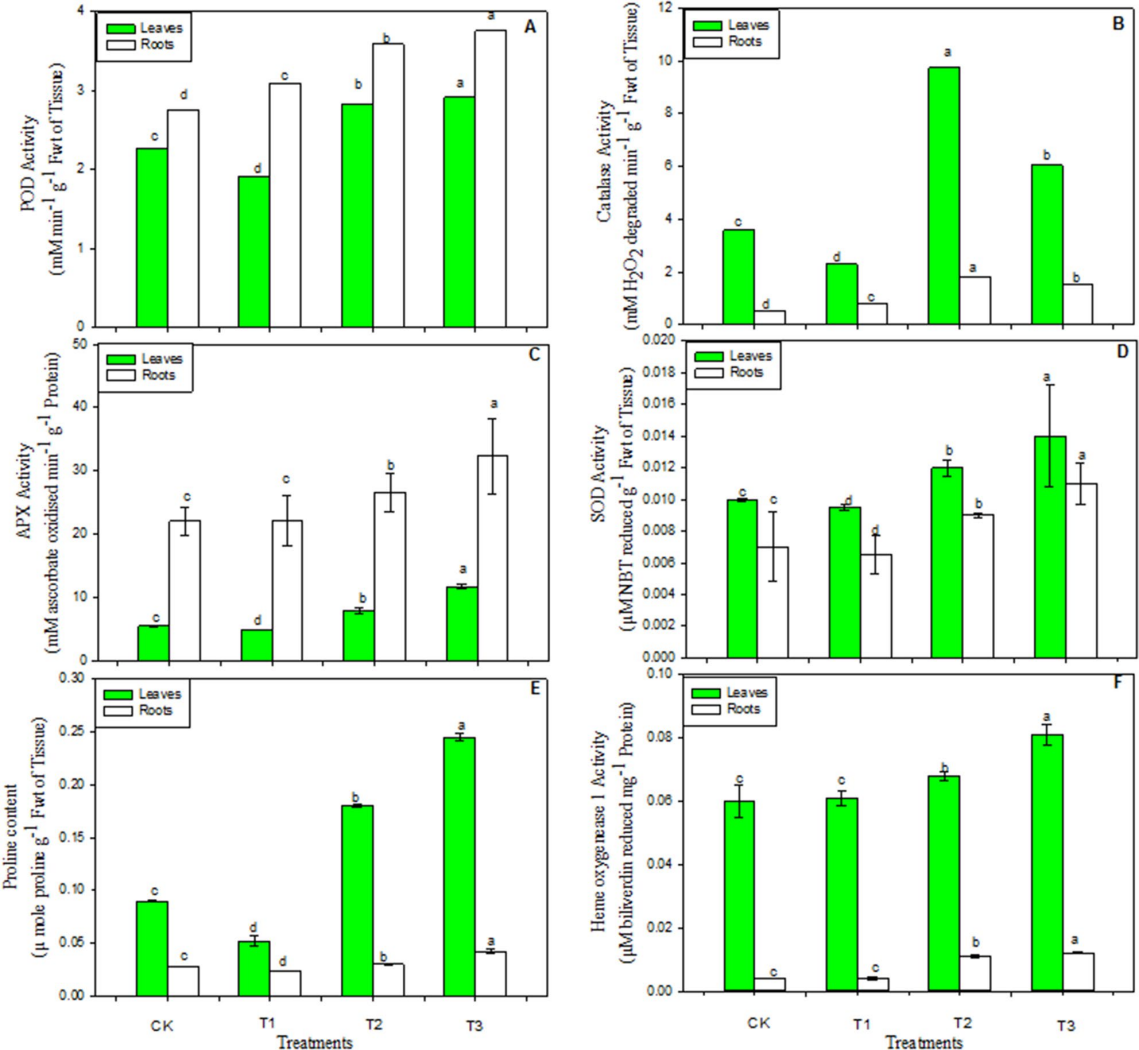
(**A**) Peroxidase (POD) (**B**) Catalase (CAT) (**C**) Ascorbate peroxidase (APX) (**D**) Superoxide dismutase (SOD) (**E**) Proline conent and **F** Haem oxygenase-1 activity in seedlings of Vigna radiata. Seedlings were treated with 50 μM CdCl_2_ independently and in combination with 0.5 mM hemin for 4 days. Values are mean ± SE of three replicates (n = 3) and are statistically significant according to DMRT test (p < 0.05). Data points marked with the same letters show insignificant differences (p < 0.05) within treatments.

To overcome the harmful effects of the cadmium treatment, plants accumulated a considerable amount of proline as a compatible osmolyte in their tissues. In the present study, under Cd stress, proline accumulation was approximately twice and 1.07 times in leaves and roots, respectively, that of control plants. However, the accretion of proline was further found to be increased by the application of exogenous hemin. In contrast to individual cadmium treatments, 0.5 mM hemin supplementation improved the proline content by 36% (leaves) and 40% (roots) in the Cd-treated seedlings of *V. radiata* (Fig. 3E).

### Activity of antioxidant enzymes

SOD catalysis increased by 20% and 28.6% in leaves and roots, respectively, after CdCl_2_ treatment in comparison to the control levels. Supplementation of 0.5 mM hemin with CdCl_2_ significantly improved SOD activity, which was 1.17 (leaves) and 1.22 (roots) times higher than that under Cd treatment alone (Fig. 3D).

Conversely, catalase activity in seedlings increased progressively upon Cd treatment, showing levels 2.71 and 3.49 times higher than those of the control. However, a sudden drop of 37.9% and 14.3% was recorded with the addition of hemin to cadmium-affected crops in comparison to the levels of plants under stress conditions only (Fig. 3B).

Peroxidase (POD) activity increased gradually by 24% (leaves) and 30% (roots) in seedlings of *V. radiata* treated with CdCl_2_ stress. However, the addition of 0.5 mM hemin resulted in a subsequent enhancement in enzyme catalysis compared to that in plants under CdCl_2_ stress alone (Fig. 3A). Similar trends of results were obtained for ascorbate peroxidase (APX). An increase in APX catalysis was recorded in both parts, with levels 1.45-(leaves) and 1.2- (roots) fold higher than those of the control upon exposure to Cd at a 50 μM concentration. Cd treatment with hemin further enhanced APX activity by 21% and 48% in roots and leaves, respectively (Fig. 3C).

The impact of various treatments on HO-1 catalysis is depicted in Fig. 3F. A considerable rise in HO-1 activity was observed upon treatment of *V. radiata* seedlings with CdCl_2_. However, the application of exogenous 0.5 mM hemin in the Cd treatment group restored HO-1 activity by 15.7% and 9.1% in leaves and roots, respectively, compared with Cd treatment alone (Fig. 3F).

### Effect of exogenous hemin on the uptake, accumulation and translocation of Cd^2+^

*Vigna radiata* seedlings treated with 50 µM CdCl_2_ showed higher accumulation of Cd^2+^ in roots than leaves, as depicted in Table 2. In terms of phytoremediation, the addition of 0.5 mM hemin progressively increased the intracellular cadmium content by 40% and 17% in leaves and roots, respectively, in comparison to those of the seedlings subjected to CdCl_2_ treatment only (Table 2).

**Table 2:** Intracellular Cd content, Biological concentration Factor (BCF) and Translocation Factor (TF) in seedlings of *V. radiata* exposed to different treatments for a period of 96 h.

Parameters	CdCl_2_ (T2)	Hemin + CdCl_2_ (T3)
Intracellular Cd content leaves (µg g^−1^ Dwt of tissue)	85.5b ± 4.0	119.8a ± 6.1
Intracellular Cd content roots (µg g^−1^ Dwt of tissue)	2478ab ± 9.3	2897a ± 10.8
Bioconcentration factor (BCF) (leaves)	0.02b ± 0.001	0.038a ± 0.001
Bioconcentration factor (BCF) (roots)	0.57b ± 0.003	0.919a ± 0.003
Translocation factor (TF)	0.035a ± 0.001	0.041a ± 0.001

Cadmium uptake from the liquid medium via roots and its translocation to the foliar tissue affected the BCF and TF. For the seedlings of *V. radiata* subjected to Cd stress, hemin increased the uptake and translocation of cadmium in the plants. Supplementation of Cd-treated plants with 0.5 mM hemin improved the BCF by 2.0-and 1.6-fold in leaves and roots, respectively. A similar pattern of results was obtained for the TF, and the transfer of Cd ions from roots to aerial parts increased by 18.8% upon the addition of hemin to the stressed plants (Table 2).

### Principal component analysis (PCA)

**Figure 4:**
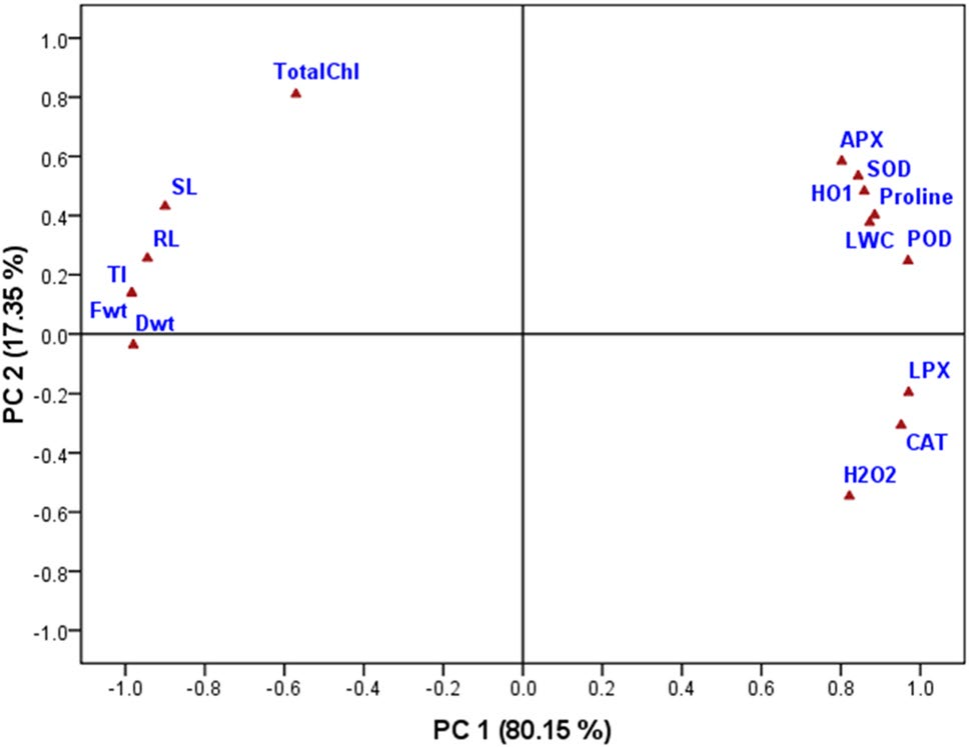
Principal component analysis of combined data sets, growth parameters: fresh weight (Fwt), dry weight (Dwt), root length (RL), shoot length (SL), leaf water content (LWC), chlorophyll content (Total Chl), stress parameters: lipid peroxidation (LPX), hydrogen peroxide content (H_2_O_2_), tolerance index and antioxidants: proline content, activity of SOD, POD, APX, CAT and HO-1 in response to various combination of treatment.

Principal component analysis was performed to determine the correlation between different evaluated parameters (morphological and physiological) in response to various treatments and to understand their importance in enhancing Cd tolerance in *V. radiata* seedlings supplemented with a low dose (0.5 mM) of hemin. The results showed that all the parameters (growth, total chlorophyll, tolerance index, stress and antioxidants) were grouped into two components (PC 1 and PC 2) and accounted for 97.5% of the total variance (Fig. 4). The first principal component showed 80.15% variance and correlated with the biomass (Fwt: fresh weight, Dwt: dry weight), plant height (SL: shoot length, RL: root length), leaf water content (LWC), total chlorophyll (Total Chl), tolerance index (TI) and antioxidants (SOD, POD, APX, proline), including HO-1. The second principal component contributed to 17.35% of the variance and was grouped with stress parameters (LPX and H_2_O_2_), the LWC and antioxidants (SOD, POD, APX, proline, HO-1, CAT) (Fig. 4).

## Discussion

As a serious environmental pollutant, cadmium causes severe damage to plants by negatively influencing its morphology and physiology^2,5,17^. The outcome of the work demonstrated that metal stress hinders overall crop development by reducing plant biomass and chlorophyll contents. However, hemin supplementation markedly alleviated the adverse impact of cadmium by improving the growth and physiology of the plants. This is in accordance with earlier reports on hemin-induced alleviation of the inhibitory impact of cadmium in *Medicago sativa* L.^11^ and *Brassica chinensis* L.^5^. The morphology of root tissues is an imperative marker to assess the toxic effects of Cd^2+^ as roots are the initial tissues that are associated with metal ions^17^. In this study, root length was significantly affected by Cd stress, which disturbs the uptake of water and nutrients via roots and reduces crop growth and biomass^5,18,19^. Thus, root length affects the uptake and translocation of Cd by plants. Increases in root length and the number of root hairs were observed with hemin supplementation, which increases the absorption of water and nutrients and ultimately enhances plant biomass. This might be because exogenous hemin advances auxin-stimulated lateral root growth, which increases root numbers by stimulating cell division^5,20^. Hence, hemin favours the uptake of nutrients and metal ions from the liquid medium^21,22^.

In addition to enhanced root growth, the impact of hemin on crop improvement was mainly reflected in the alterations in photosynthetic pigments, as photosynthesis is the basis of plant growth and development. In our study, the chlorophyll content noticeably declined upon exposure of crop seedlings to Cd stress, which is probably due to damage to the photosynthetic apparatus, specifically photosystems I and II^5,23^. Cadmium blocks the photoactivation of photosystems by hindering electron transport in plastids^24^. Moreover, Cd inhibits the Calvin cycle (an important cycle of the dark reaction during photosynthesis) by affecting enzyme activities, which results in a lower photosynthetic rate^25^. However, hemin supply progressively improved the chlorophyll content in the stressed seedlings. The role of hemin in improving the chlorophyll concentration might be correlated with augmented haem oxygenase-1 activity. The results of this study are comparable with prior research results that demonstrated the role of haem oxygenase as an essential element in chlorophyll biosynthesis^26–29^.

The cadmium concentration in liquid medium changes the osmotic balance of plant cells by hindering water uptake through roots and reducing the leaf water content (LWC)^2^. Conversely, our study showed no noticeable reduction in the leaf water content under Cd stress, which might have been due to the progressive increase in the proline content in *V. radatia* seedlings. Proline is an effective quencher of ROS and works as a metal chelator in stress environments^30,31^. The addition of hemin to Cd-stressed plants further improved the proline content, which restored the leaf water content in plants. The improvement in the LWC due to the increased proline content was probably due to the antioxidant nature of proline, which alters plant metabolic activities to improve stress tolerance^31,32^. A simultaneous increase in the proline content was reported earlier in different plant species subjected to Cd stress^2,33–35^.

The toxic effects of cadmium in the present work were indicated by the overproduction of H_2_O_2_ and increased accumulation of MDA. The MDA content, a product of lipid peroxidation, is considered a signal of membrane destruction^27^. The study outcomes demonstrate an increase in toxicity in seedlings of *V. radiata* subjected to cadmium stress. Parallel effects of Cd in other plant species were also reported in earlier studies^2,5,33,34^. However, addition of hemin to metal-treated crops reduced the detrimental effect of cadmium in the seedlings and enhanced their physiology. These study results were in accordance with those of Zhu et al*.*^5^, who determined the mitigation of cadmium stress in *Brassica chinensis* L. with an application of exogenous hemin to the treated crop. The shielding impact of hemin might be associated with the increased activities of antioxidants, which were probably mediated by haem oxygenase-1^11^.

SOD, CAT, APX and POD directly participate in ROS detoxification. ROS detoxification commences with the catalytic reaction of superoxide dismutase, the preliminary antioxidant that scavenges ROS by converting O_2_^-^ to H_2_O_2_ and O_2_^36^. Further H_2_O_2_ is converted to water through CAT, POD and APX. The accumulation of H_2_O_2_ inside the plant cell is prohibited by CAT, while APX and POD reduce it to H_2_O so that ROS deposition is efficiently controlled^37^. These antioxidant enzymes show regular activity under normal conditions, but their catalytic reaction is magnified under stress^17^. In our study, the activity of all antioxidants, including that of SOD, CAT, POD and APX, increased under Cd stress, which shows the clear response of *V. radiata* seedlings to metal stress and is likely one of the reasons for their survival along with Cd accumulation. Similar trends in antioxidant activity were recorded in different plant species exposed to Cd stress^2,9,17,33,34^.

However, exogenous application of 0.5 mM hemin further augmented the catalysis of APX, SOD and POD, which decreased the ROS concentration in *V. radiata* seedlings. These findings are comparable with those of Zhu et al*.*^5^, which verified the positive effect of hemin on antioxidant enzyme activity in seedlings of *Brassica chinensis* L. treated with Cd stress. Similarly, Chen et al*.*^38^ reported that supplementation with 1 μM and 5 μM hemin enhanced SOD and APX activity in *Oryza sativa* L. seedlings exposed to Zn (1 μM), Pb and Cr (5 μM)-treated hydroponic medium. The affirmative impact of hemin on antioxidant activities might be associated with the role of haem oxygenase-1. Hemin is a potent stimulator of HO-1^39^, which oxidizes haem to biliverdin (BV), Fe^2+^ and carbon monoxide by employing NADH as a reducing equivalent^40^. Haem oxygenase-1 maintains the normal physiology of plants under stress environment by regulating antioxidant activities which neutralizes the negative effect of metal stress^17,41^. In recent findings, supplementation of 0.5 mM hemin to the Cd stress medium up-regulates the activity of haem oxygenase-1 enzyme to many folds (1.35 folds higher than control) which induces the activities of APX, SOD, POD and proline. The stimulated activity of antioxidants (APX, SOD, POD and proline) by HO-1 was clearly apparent in Fig. 4 which shows a linear co-relation with HO-1. The highest correlation of HO-1 was recorded with SOD and APX (Fig. 4). Additionally, CAT, POD and APX are haem enzymes with Fe in their structures, so Fe deficiency (due to competition between Cd and bivalent ions) is probably a limiting factor for their activities under Cd stress^3^. Hemin application in the Cd-treated medium elevates the activity of these antioxidants by increasing accessibility to iron (formed as a by-product during haem catalysis by HO-1)^17^. The study is supported by numerous reports that validate the shielding action of haem oxygenase towards cadmium treatment in different plant genotypes^17,41–43^. Furthermore, the PCA results revealed that augmented antioxidant activity improved the harvestable biomass and chlorophyll content of *V. radiata* under the combined treatment, and these parameters were negatively correlated with the H_2_O_2_ content and membrane damage (LPX) (Fig. 4).Thus our findings display the exact mechanism of hemin to tolerate Cd^2+^ by intensifying the activities of antioxidants which is mediated by hemin induced upregulated activity of HO-1.

To understand the uptake and translocation behaviour of metals in plants, it is important to select a suitable agent for improving the phytoremediation and stress tolerance of plants. Hemin, as a biostimulator, is known to enhance stress resilience in crops^5,38^. Similar to the results from other prior studies, the intracellular cadmium concentration in our study was far higher in roots than in leaves^3,17,33,34^. However, the intracellular Cd concentration in both tissues, viz. leaves and roots, was much lower than the critical concentration reported from Cd hyperaccumulators, which is 100 mg kg^−1^ dry weight of tissue^3^. Moreover, the BCF of leaves and roots as well as the TF were also less than 1. Thus, in our study, *Vigna radiata* did not display the characteristics of a cadmium hyperaccumulator according to the standard described by Baker and Brooks^44^ but was considered a cadmium eliminator that efficiently removes Cd from the contaminated environment.

In the current investigation, BCF and TF declined upon exposure of *V. radiata* seedlings to cadmium, as revealed by Mahmud et al*.*^2^ and Nabei and Amooaghaie^3^ in *Brassica juncea* L. and *Catharanthus roseus* (L.) G. Don; treated with Cd concentrations. This decrease may be due to the reduction in biomass and chlorophyll content of seedlings. Moreover, the decline can also be attributed to the saturation of metal uptake and root-to-leaf translocation^45^. Interestingly, exogenous application of hemin at a lower dose (0.5 mM) enhanced metal accretion in *V. radiata* tissues, which confirmed the efficiency of hemin for phytoremediation. Correspondingly, the uptake and transfer of metal from roots to leaves was noticeably amplified upon supplementation of hemin to Cd-stressed medium (Table 2). The effective promotion of the BCF and TF with the application of hemin was due to many reasons. The first probable reason is that hemin promotes auxin (specifically IAA)-induced lateral root development, which has already been reported in several studies conducted on different plant species^46–49^. Increased production of endogenous auxin stimulates the activity of membrane H^+^ ATPases that alter the transport of cation transporters^50^ and advances the absorption of Cd by *V. radiata*. A similar increase in the efficient absorption of Cd and U ions by an exogenous treatment of IAA was reported by Chen et al*.*^9^ in *B. juncea*. Moreover, auxin increases the solubility and bioavailability of Cd in the growing medium by decreasing the pH of the medium, which enhances metal absorption via roots and transfer to aerial tissues^9,26^. Second, hemin supplementation of Cd-stressed plants triggers a haem oxygenase-1 mediated antioxidant defence mechanism that improves the endogenous concentration of Fe^2+^ and CO (by-products of the HO-1 catalytic reaction) in the liquid medium. Improved Fe^2+^ and CO concentrations increase the uptake and accumulation of Cd while decreasing micronutrient uptake in plant tissues^51^. This might be understood on the basis of competition between Cd and micronutrients, particularly bivalent ions (Fe, Mg, Zn, Cu and Ca), for the transport channels that are involved in the translocation of these ions^3^.

Additionally, in the present study, hemin increased the tolerance index along with Cd accumulation in plant tissues, which suggests that the defensive role of hemin is not associated with the inhibition of uptake and transfer of metals but is possibly involved in activating the endogenous mechanism of cadmium detoxification in *V. radiata*. This assumption was subsequently verified by the finding that the application of hemin increased Cd translocation to the foliar parts, and despite the higher Cd concentration in the aerial tissues, the growth and physiology of the plant improved. This might be due to the release of some strong ligands by hemin to the Hoagland medium, which counterbalanced the Cd ion concentration in the plants. Moreover, hemin may also act as a metal chelator and efficiently translocate Cd over long distances through the xylem to avoid the high toxicity of Cd ions in the edible parts of plants, similar to other biostimulators^3^. Our results are in contrast with previous reports on Chinese cabbage^5^, rice^38^ and alfalfa^11^, where hemin application inhibited metal uptake and accumulation in plants to mitigate the noxious effects of cadmium ions. These discrepancies in the results are probably because the impact of hemin cotreatment on heavy metal accumulation depends on the type of plant species, concentration of the metal and hemin, exposure time and experimental conditions. Additionally, hemin might also increase metal accumulation in tolerant plants while decreasing metal accumulation in vulnerable species^7,52^ which is why diverse effects were observed based on the physiological nature of the plants.

## Conclusion

The present investigation explores the role of hemin application in the advancement of Cd stress tolerance and remediation efficiency using *Vigna radiata*. Cadmium adversely affects plant growth by reducing the harvestable biomass and chlorophyll content and increasing oxidative stress (H_2_O_2_ and MDA content). However, exogenous supplementation of hemin in liquid medium that also contains Cd mediates the initiation of tolerance mechanisms in plants through upregulation of antioxidant activities. Moreover, this study presented strong evidence that hemin supports the removal efficiency of *V. radiata* by (1) improving the plant biomass and chlorophyll content and (2) enhancing Cd uptake and its translocation from roots to foliar tissues. Thus, our data concluded that hemin, as a biostimulator, has the potential to improve the phytoremediation efficiency of heavy metal tolerant plants so that they can be used as an alternative to hyperaccumulators to remediate Cd contamination. Future studies in the natural environment need to be carried out to further verify the significance of hemin for cadmium tolerance and remediation.

## Materials and methods

### Processing of experimental material and stress treatments

*Vigna radiata var. PDM 54* seeds were collected from the National Bureau of Plant Genetic Resources, Jodhpur, India and disinfected with mercuric chloride (0.1% w/v) for one minute followed by rinsing with autoclaved deionized water (4–5 times) to remove the remaining traces of HgCl_2_. Disinfected seeds were germinated in sterile conditions in petri-plates with blotting paper soaked with distilled water at room temperature in the dark in a seed germinator. At the two-leaf stage, the germinated seedlings were transferred to half-strength Hoagland medium (pH 6.8–6.9) and placed under thermostatically controlled conditions (50% relative humidity and 25 ± 2 °C temperature). The liquid medium was replaced with fresh medium every other day and aerated twice daily to avoid nutrient and oxygen deficiency in the seedlings.

**Figure 5:**
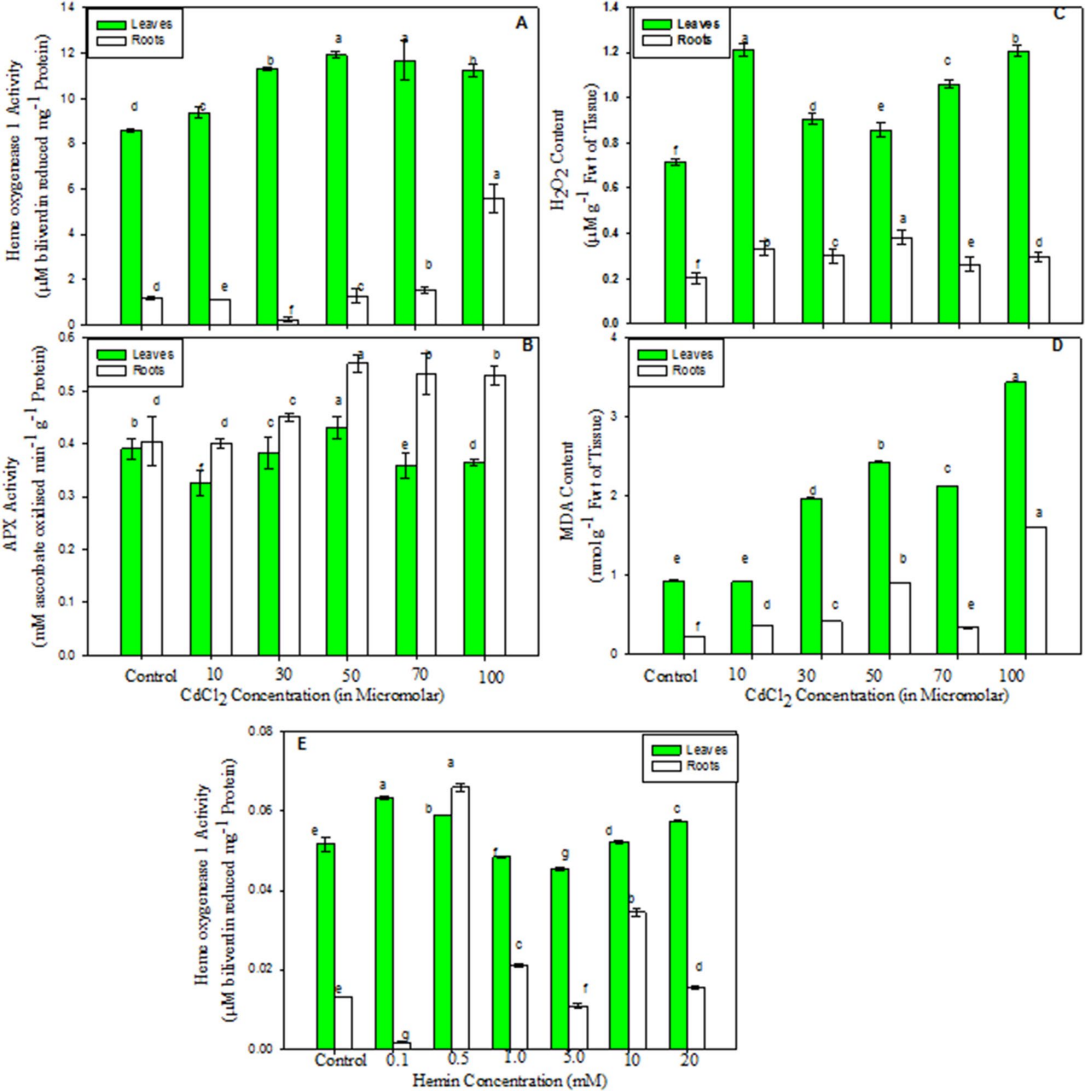
Dose dependent effect of CdCl_2_ (10 μM to 100 μM) (from **A** to **D**) and hemin (0.1 to 20 mM) (**E**) on antioxidants activity: HO-1 (**A** & **E**) and APX (**B**); Oxidative damage: H_2_O_2_ (**C**) and MDA content (**D**) in *Vigna radiata*. Seedlings were treated independently for a period of 96 h to select the optimum concentration of CdCl_2_ and hemin for further analysis. Values are mean ± SE (n = 3) and are statistically significant according to DMRT test (p < 0.05). Data points marked with the different letters show significant differences (p < 0.05) within treatments.

After 1 week, seedlings successfully adapted to hydroponic culture were treated with exogenous hemin at concentrations ranging from 0.1 to 20 mM (0.1, 0.5, 1.0, 5.0, 10, 20 mM) for 96 h^53–57^. Liquid medium without treatment was considered a control and utilized to evaluate the effect of hemin on haem oxygenase-1 catalytic activity. Maximum haem oxygenase-1 (HO-1) catalysis was observed under the 0.5 mM treatment (Fig. 5E); hence, this hemin concentration was selected for further analysis. Based on our previous report on the role of HO-1 in mitigating Cd stress in *V. radiata* seedlings^17^, we selected a 50 µM CdCl_2_ concentration for the present study because at this treatment level, the activities of all the antioxidants, including haem oxygenase-1, were maximal (Fig. 5A,B), which resulted in the lowering of oxidative damage at this concentration (Fig. 5C,D). Thus, the seedlings of *V. radiata* used in the present study were subjected to the following treatments:

1. Hoagland nutrient solution without any treatment (Control, CK)
2. 0.5 mM hemin (T1)
3. 50 µM CdCl_2_ (T2)
4. 50 µM CdCl_2_ + 0.5 mM hemin (T3)

The treated plants were harvested after 96 h of stress for assessment of different physiological parameters^17^.

### Morphological parameters

To study morphological parameters, freshly harvested crop seedlings were first washed with distilled water. Crop morphology was examined with regard to seedling height (root and shoot length), biomass (fresh and dry weight), a tolerance index (TI) and the leaf water content (LWC). The height of the seedlings was individually computed in centimetres. To calculate the dry weight, fresh seedlings were desiccated overnight at 65 ˚C in an oven, and the weight of dehydrated seedlings was measured. The TI was measured according to the Wilkins^58^ method and presented as percent tolerance. The LWC was calculated from seedling biomass by utilizing the equation (fresh weight − dry weight/fresh weight) × 100^59^.

### Quantification of chlorophyll concentrations

Chlorophyll concentrations (Chl a, Chl b and total Chl) were quantified by the Arnon^60^ protocol. Freshly harvested young leaves were pulverized in 80% chilled acetone at 4 °C and centrifuged for 15 min at 10,000×*g* under cold conditions. The chlorophyll content (mg g^−1^ fresh weight of leaves) was determined from the optical density of the procured supernatant recorded at 645 nm and 663 nm^60^.

### Determination of oxidative stress

#### Peroxidation of membrane lipids

The peroxidation of membrane lipids was determined by quantifying the malondialdehyde (MDA) content according to the protocol of De Vos et al*.*^61^. Freshly harvested seedlings were crushed in 2-thiobarbituric acid (0.25% w/v) prepared in trichloroacetic acid (10% v/v). The ground sample was incubated for 30 min at 95 °C in a water bath and centrifuged for 15 min at 10,000×*g* after cooling to room temperature. The MDA concentration (nanomolar per gram fresh weight of tissue) was quantified from the specific optical density (λ_532_–λ_600_) by utilizing 155 mM^−1^ cm^−1^ as a proportionality constant^61^.

### H_2_O_2_ concentration

Fresh plant tissues were homogenized in TCA (0.1% v/v) under cold conditions. The pulverized sample was centrifuged for 15 min at 10,000×*g*. The H_2_O_2_ concentration (µM g^−1^ fresh weight of tissue) was determined by documenting the optical density of an assay compound [potassium phosphate buffer (10 mM, pH 7.0) + KI (1 M) + supernatant] incubated in the dark for an hour at 390 nm (molar absorption coefficient 0.28 µmol^−1^ cm^−1^)^62^.

### Estimation of proline content

The proline content was determined by the procedure of Bates et al*.*^63^. Freshly harvested tissues were extracted in sulphosalicylic acid (3% w/v) and subsequently centrifuged for 20 min at 3000×*g*. The reaction mixture (equivalent volume of acid ninhydrin + supernatant + glacial acetic acid) was incubated for an hour at 60 °C in a water bath and transferred to an ice bath to terminate the reaction. The proline concentration (µg g^−1^ fresh weight of tissue) was estimated by documenting the optical density of an organic chromophore at 520 nm after adding toluene to the terminated reaction mixture^63^.

### Extraction and assay of antioxidant enzymes

Freshly harvested crop seedlings were pulverized in NaPO_4_ buffer (50 mM, pH 7.0) in cold conditions. The homogenate was centrifuged for 20 min at 10,000×*g* at 4 °C. The procured supernatant was utilized for further analysis.

Superoxide dismutase (SOD) catalysis was assayed by the Beuchamp and Fridovich^64^, protocol. The catalytic reaction of SOD was determined by documenting the optical density of the assay compound [NaPO_4_ buffer (50 mM, pH 7) + methionine (13 mM) + nitroblue tetrazolium (NBT) (75 µM) + riboflavin (2 mM) + EDTA (0.1 mM) + enzyme extract] at 560 nm after 30 min of incubation under bright light, which indicates the capability of an enzyme to hinder the photolytic reduction of NBT (with a proportionality constant of 100 mM^−1^ cm^−1^).

The catalytic reaction of the catalase (CAT) enzyme was measured via the Aebi^65^, procedure. The decomposition rate of H_2_O_2_ (mM H_2_O_2_ degraded min^−1^ g^−1^ fresh weight of tissue) was evaluated by the decline in optical density of the assay mixture [NaPO_4_ buffer (50 mM, pH 7) + H_2_O_2_ (9 mM) + enzyme extract] at 240 nm by utilizing 0.039 mM^-1^ cm^-1^ as the molar absorption coefficient^65^.

The catalysis of ascorbate peroxidase (APX) was determined via Chen and Asada^66^. The oxidation rate of ascorbate (mM ascorbate oxidized min^−1^ g^−1^ fresh weight of tissue) was computed by the decline in optical density of an assay compound [NaPO_4_ buffer (50 mM, pH 7) + H_2_O_2_ (10% v/v) + enzyme extract + ascorbate (0.6 mM)] at 290 nm by employing 2.8 mM^−1^ cm^−1^ as a proportionality constant^66^.

The peroxidase (POD) catalysis was measured by using the method of Putter^67^. The rate of formation of tetraguaiacol (mM min^−1^ g^−1^ fresh weight of tissue) was calculated by the rise in optical density of an assay mixture [NaPO_4_ buffer (50 mM, pH 7) + guaiacol (20 mM) + H_2_O_2_ (3.7 mM) + enzyme extract] at 436 nm by employing 26.6 mM^–1^ cm^–1^ as a proportionality constant^67^.

The catalysis of the haem oxygenase-1 (HO-1) enzyme was evaluated according to the Balestrasse et al*.*^68^ method. Freshly harvested plant tissue was homogenized in chilled KPO_4_ buffer (50 mM, pH 7.4) containing PMSF (1 mM) + EDTA (0.2 mM) + sucrose (0.25 M). The procured supernatant, after centrifugation of the homogenate for 20 min at 10,000×*g* at 4 °C, was employed for HO-1 analysis. The concentration of HO-1 (µM biliverdin reduced mg^−1^ protein) was estimated by documenting the optical density of biliverdin (main product of HO-1catalysis) formed by the reaction between an assay compound [KPO_4_ buffer (50 mM, pH 7.4) + hemin (200 nm) + NADPH (60 nm)] and HO-1 extract at 37 °C for one hour at 650 nm (Molar absorption coefficient 6.25 µM^–1^ cm^–1^).

### Intracellular Cd concentration

The Cd content accumulated in the plant cells was determined via the protocol of Bates et al*.*^69^. Fresh plant samples were cleansed with distilled water, and roots were separated from the aerial tissue. Separated tissues were kept in an oven overnight at 80 °C. Desiccated tissues were boiled in an acid mixture containing HNO_3_ (70% v/v) + H_2_O_2_ (30% v/v) + deionised water in the proportion of 1:1:3. The colourless remains of the mixture were diluted in HNO_3_ (2% v/v) and employed for estimation of the Cd content^69^.

### Measurement of biological concentration factor (BCF) and translocation factor (TF)

The biological concentration factor reveals the ability of corresponding plant species to uptake the particular metal ion into tissues to its proportion in the related surroundings and was calculated by the formula^17^:

$${\text{BCF}} = \frac{{\left[ {{\text{Metal}} \,\upmu {\text{g}} \,{\text{g}}^{ - 1} \,{\text{DW}}} \right]_{{\left( {{\text{root}}/{\text{leaf}}} \right)}} }}{{\left[ {{\text{Metal}} \,\upmu {\text{g}} \,{\text{L}}^{ - 1} } \right]_{{\left( {{\text{nutrient}}\,{\text{ solution}}} \right)}} }}.$$

The translocation factor demonstrates the transfer ability of metal ion in particular plant^70^. The formula for estimating TF is:

$${\text{TF}} = \frac{{\left[ {{\text{Metal}}\,\upmu {\text{g }}\,{\text{g}}^{ - 1} { }\,{\text{DW}}} \right]_{{\left( {{\text{leaf}}} \right)}} }}{{\left[ {{\text{Metal }}\,\upmu {\text{g g}}^{ - 1} { }\,{\text{DW}}} \right]_{{\left( {{\text{root}}} \right)}} }}.$$

### Statistical analysis

For graphical representation of data Sigma plot version 12.0 (Chicago, IL, USA) software (http://www.sigmaplot.co.uk/products/sigmaplot/produpdates/prod-updates5.php) was used. Data were statistically examined by one way ANOVA and the correlation between different evaluated parameters were analyzed by principal component analysis using SPSS 16 version software. The data were considered as average (± standard error) of individual replicas (n = 3) of every test conducted separately. To check the reproducibility of results, three independent biological replicates were studied following completely randomized design (CRD). The significant variations among treated and untreated seedlings were illustrated at 0.05% significant level by applying Duncan’s Multiple Range test (DMRT)^17,59^.
